# 

**Published:** 1887-01-01

**Authors:** 


PRICE ONE PENNY.

No. 14, Vol. I.

January 1st, 1887.

Per Annum, 6s. 6d., post free.
Monthly Parts, 6d.; post free, 7½d
Ditto per Ann., 7s. 6d., post free.

